# Evaluation of clinical cardiac safety of zilurgisertib, an activin receptor‐like kinase‐2 (ALK2) inhibitor, in healthy participants

**DOI:** 10.1002/cpdd.1618

**Published:** 2025-10-23

**Authors:** Yan‐ou Yang, Hong Yang, Xing Liu, Xiaohua Gong, Jay Getsy, Kevin Rockich

**Affiliations:** ^1^ Clinical Pharmacology and Pharmacometrics Incyte Corporation Wilmington Delaware USA; ^2^ Early Clinical Development Incyte Corporation Wilmington Delaware USA

**Keywords:** activin, ALK2 inhibitor, C‐QTc analysis, QTcI, ECG

## Abstract

The oral, small molecule inhibitor of activin receptor‐like kinase‐2, zilurgisertib (INCB000928), is under evaluation in fibrodysplasia ossificans progressiva. Cardiac safety was assessed using electrocardiogram (ECG) parameters and a plasma concentration‐heart rate‐corrected QT (C‐QTc) interval analysis of pooled data from single ascending dose (SAD) and multiple ascending dose (MAD) studies of zilurgisertib in healthy adult participants (SAD: 10‐500 mg; INCB00928‐102: 50‐400 mg QD, 300 mg BID). Overall, 91 (SAD) and 79 (MAD) participants provided at least one pair of PK/ECG data. As both studies indicated a dose‐dependent effect of zilurgisertib on heart rate, individualized QT correction (QTcI) was used as the primary endpoint for QTc analysis. Estimated population slope of the individualized C‐ΔQTc (C‐ΔQTcI) relationship was shallow (0.02 ms per µm [90% CI, −0.60, 0.65]) and not statistically significantly different from 0; treatment effect–specific intercept was small and not significant (−0.83 ms [90% CI, −2.26, 0.61]). No significant relationship between zilurgisertib plasma concentration and change in QTcI was identified; zilurgisertib did not have a clinically relevant effect on QTc prolongation. QT effect *>*10 ms could therefore be excluded within the dose range studied (up to 300 mg BID). No clinically meaningful effects on cardiac conduction (PR and QRS intervals) or any categorical PR or QRS outliers were observed. These data support further clinical development of zilurgisertib.

Zilurgisertib (INCB000928) is an orally administered small molecule inhibitor of activin receptor‐like kinase‐2 (ALK2).^1^, ^2^ ALK2 belongs to the bone morphogenetic protein (BMP) family of receptors that mediates activation of SMAD1/5/8 signaling and modulates expression of several genes.^3^ In common with other BMP receptors, ALK2 activates osteogenic signaling and plays an important role in the development and repair of bone and cartilage. ALK2 is also a central player in regulating hematopoiesis and iron homeostasis.^3^, ^4^

Mutations and/or altered expression levels in ALK2 are associated with several human disorders, such as fibrodysplasia ossificans progressiva (FOP), diffuse intrinsic pontine glioma, and diffuse idiopathic skeletal hyperostosis.^3^, ^4^ Almost all patients with FOP carry an *ALK2* mutation (R206H) that results in dysregulated BMP signaling, which plays a major role in the development and progression of heterotopic ossification.^4^, ^5^ Zilurgisertib is currently being evaluated in a phase 2 pivotal study for the treatment of patients with FOP (NCT05090891).

In keeping with the International Conference on Harmonisation of Technical Requirements for Pharmaceuticals for Human Use (ICH) E14 guidelines and the methodologies previously described for concentration–heart rate (HR)‐corrected QT interval (C‐QTc) modeling,^6^ the cardiac safety analysis of zilurgisertib was evaluated during the drug development process. In a patch‐clamp assay, half‐maximal inhibitory concentration for human Ether‐à‐go‐go‐Related Gene (hERG) inhibition was estimated to be >100 µm (or 50 µg/mL) (Data on file, Incyte Corporation). In addition, biochemical and cellular assays showed that zilurgisertib was selective to ALK2 inhibition, potentially minimizing off‐target effects. Minimal off‐target effects were also indicated by data showing that zilurgisertib did not affect viability of human embryonic kidney 293 cells, a human cell line commonly used to assess general cell health and compound toxicity, and did not affect viability of human fibroblasts or endothelial cells in concentrations up to 5 µm (or 2.5 µg/mL).^1^, ^7^

Clinical pharmacokinetics (PK) of zilurgisertib have been previously reported for both a single‐ascending dose study (SAD; INCB00928‐101) and a multiple‐ascending dose study (MAD; INCB00928‐102).^8^ Briefly, following single oral doses (10‐500 mg) in the SAD study, zilurgisertib was rapidly absorbed with a geometric mean of maximum plasma drug concentration (C_max_) of 2460 nm (or 1237 ng/mL) for the 500 mg dose; median time to maximum concentration (t_max_) in plasma was reported as 2.0‐4.1 hours and zilurgisertib exposure increased approximately dose proportionally. Data indicated that zilurgisertib can be administered without regard to food. Following multiple oral dose administrations (50‐400 mg once daily [QD] and 300 mg twice daily [BID]) in the MAD study, median plasma t_max_ was 2.0‐4.0 h, with geometric mean C_max_ values of 4250 nm (or 2138 ng/mL) for 400 mg QD and 4690 nm (or 2359 ng/mL) for 300 mg BID, respectively. Plasma half‐life values ranged from 22.8 to 27.5 hours, and steady state was reached by day 5. Zilurgisertib exposure increased modestly more than dose proportionally, with ∼1.9‐ to 2.6‐fold accumulation in plasma area under the curve with QD dosing and ∼3.8‐fold accumulation with 300‐mg BID dosing.

The objective of this analysis was to evaluate the effect of zilurgisertib on QT intervals using a model based approach, as well as to assess other electrocardiogram (ECG) parameters, based on PK and ECG data collected during the phase 1 SAD and MAD studies in healthy adult participants.^8^

## Methods

### Ethics

Before study initiation, the institutional review board (Advarra Inc., Columbia, MD, USA) approved the study protocols and the ethical, scientific, and medical appropriateness of the study; all participants provided written informed consent. Studies were conducted in accordance with ethical principles based on the Declaration of Helsinki, International Council for Harmonisation Guideline for Good Clinical Practice, and applicable laws and regulations.

### Participants and study design

Healthy adult participants, aged 18‐55 years, were enrolled across eight cohorts in the SAD study and six cohorts in the MAD study as previously described.^8^ In the SAD study, single ascending doses of zilurgisertib at 10, 25, 50, 100, 175, 250, and 500 mg or placebo were administered orally to 79 healthy adult participants enrolled in seven cohorts (cohorts 1‐7). Food effect was studied in 12 participants in cohort 9, in which bioavailability of a single oral dose of zilurgisertib 100 mg in the fed (high‐fat meal) and fasted states were evaluated. In the MAD study, multiple ascending doses of zilurgisertib at 50, 100, 150, 200, and 400 mg or placebo were administered orally once daily (QD) to 60 healthy adult participants enrolled in five cohorts (cohorts 1‐5), and 300 mg or placebo was administered twice daily (BID) to 19 participants in cohort 6.

### Study assessments

#### Pharmacokinetics

Blood samples for PK analysis were collected as follows: SAD study: 0 hour (pre dose) and 0.5, 1, 2, 4, 6, 8, 12, 16, 24, 36, 48, 72, 96, 120, and 144 hours post dose; MAD study: 0 hour (pre dose) and 0.5, 1, 2, 4, 6, 8, 12, and 16 hours (for cohorts 1‐5 only) post dose on day 1; pre dose on day 2 (i.e. 24 hours after the morning dose on day 1); 0 hour (pre dose) and 0.5, 1, 2, 4, 6, 8, 12, and 16 hours post dose on day 10; and pre dose on day 11 (i.e. 24 hours after the morning dose on day 10). Methodology for analysis of PK parameters has been described previously by Yang et al., with plasma sample collection and analysis methodology available in the Supplementary Methods.^8^

#### ECG assessment

Change from baseline (Δ) in QTc by Fridericia's method (ΔQTcF) was measured using ECG monitoring by 12‐lead Holter recording, based on the assumption that there was no drug effect on HR; if a drug effect was observed, QTcs would be assessed by an alternative method. Other ECG parameters evaluated included Δ in HR, the PR interval, and QRS interval; placebo‐adjusted Δ (ΔΔ) in QTc, HR, PR, and QRS; and categorical outliers for QTc, ΔQTc, HR, PR, and QRS. ECG was performed continuously from 4 hours pre dose to 24 hours post dose on day 1 (all cohorts) and day 8 (cohort 9 only) in the SAD study, and from 4 hours pre dose through 24 hours post dose on days 1 and 10 in the MAD study. ECG intervals were measured by a central laboratory (ERT Inc., Pittsford, NY) in a blinded manner using the Expert Precision QT (EPQT) technique,^9^ which involved extracting three replicate 10‐second ECGs per timepoint from continuous Holter recordings. Baseline and on‐treatment ECGs for a particular participant were overread on the same lead and were analyzed by the same reader. A trained ECG analyst and cardiologist reviewed all ECGs.

#### Time‐matched mapping between PK measurements and ECG assessments

Mapping of time points for C‐QTc analysis was set as follows: means of the ECG readings extracted from the continuous Holter recordings (three to ten readings per time point) across approximately 30, 60, and 90 min before study dose administration were used as baseline ECG data. For the food effect cohort 9 in the SAD study, means of the pre‐dose ECG data per treatment period were derived and used as the baseline ECG. Mean ECG readings extracted from continuous Holter recordings at ∼5‐15 min earlier than the actual PK sampling time were used as time‐matched ECG data. Means of the pre‐dose ECGs on day 10 in the MAD study were derived and regarded as on‐treatment ECGs, whose Δ values were derived with respect to the baseline ECG on day 1.

### Statistical methods

All statistical analyses were performed using SAS^®^ v9.4 (SAS Institute Inc., Cary, NC). Continuous data were summarized using descriptive statistics including number of participants, mean, median, standard deviation, standard error, 90% CI, minimum, and maximum by treatment/dose, study day, and timepoint. In all analyses, concentrations that were below the limit of quantification were entered as 0.

#### Analysis populations

Safety analyses were conducted in the safety population, which comprised all randomized participants who took at least one dose of study drug (zilurgisertib or placebo). PK analyses were conducted in the PK‐evaluable population, which included randomized participants who took at least one dose of zilurgisertib and had at least one measurable PK sample collected. Categorical and central tendency analyses were conducted in the QT/QTc population, comprising all participants in the safety population with continuous ECG measurements at baseline and on treatment. The C‐QTc analysis was conducted in the PK/QTc population, which comprised all participants who were in the QT/QTc population and the PK‐evaluable population plus participants treated with placebo with at least one pair of time‐matched post‐baseline PK and QTc data. The PK/QTc data from cohort 9 of the SAD study (12 participants fed with high‐fat meals) were excluded from the ECG central tendency analysis and the primary C‐QTc analysis but were analyzed in the sensitivity analysis of food effects on QTc shortening.

##### ECG parameters

The default HR correction method was QTcF, defined as:

$$\;\mathrm{QTc}F_{i},j={\mathrm{QT}}_{i},j/{{\mathrm{RR}}_{i},j}^{1/3}$$

for participant i at time point j, in which the time elapsed between two consecutive R waves (RR). Interval is in the unit of seconds.

However, as described in the results, dose‐dependent HR increases were observed. As such, individualized QTc (QTcI) was derived in which a linear mixed effect (LME) model was used to estimate the individual slope (i.e. the individual HR correction exponent index).

All the time‐matched QT‐RR data pairs collected under the drug‐free condition (4‐hours pre‐dose period on day 1) were fitted to an LME model. These data were comprised of all the individual/raw measurement of 12‐lead ECG interval at pre‐dose of the first treatment period for each participant.

The individualized heart rate corrected QT interval were derived as follows:

$$\mathrm{QTc}I_{i},j=QT_{i},j/R{R_{i},j}^{\left({\overset{⌢}{\beta }}_{0}+{\overset{⌢}{\beta }}_{i}\right)}$$

for participant i at time point j, in which ${\hat{\;\beta }}_{0}$ is the best linear unbiased estimation (BLUE) of the fixed effect slope parameter and ${\hat{\;\beta }}_{i}$ is the best linear unbiased prediction (BLUP) of the random effect slope parameter for participant i.

Δ in QTcI (ΔQTcI) for participant i at time j was derived as:

$$\Delta \mathrm{QTc}I_{i,j}=\mathrm{QTc}I_{i,j}\;-\mathrm{QTc}I_{i0}$$

where $\mathrm{QTc}I_{i0}$ stands for baseline QTcI for participant i.

ΔΔ in QTcI (ΔΔQTcI) for participant i at time j under active treatment of zilurgisertib at a given dose level was derived for each participant as:

$$\Delta \Delta {\mathrm{QTcI}}_{i,j}=\Delta Q{\mathrm{TcI}}_{i},j\left(\mathrm{Dose}\right)-\Delta Q{\mathrm{TcI}}_{j}\left(\mathrm{Plac}\mathrm{ebo}\right)$$

where $\Delta Q{\mathrm{TcI}}_{j}\left(\mathrm{Plac}\mathrm{ebo}\right)$ is the model‐based prediction of mean ΔQTcF at time j for the treatment of placebo.

Δ and ΔΔ for other ECG parameters (HR, PR, and QRS) were derived in the same way.

### Categorical analysis

Analysis results for categorical outliers were summarized in frequency tables, with counts and percentages for both number of participants and number of observations. A participant or observation was determined as an outlier if specified criteria (which were assessed separately) were met for the ECG intervals (Table S1). Means of ECG and nominal times were used in all cases.

### Central tendency analysis

The central tendency analysis for ΔQTc was based on a linear mixed effects (LME) repeated measures model, with ΔQTc as the dependent variable; nominal time (categorical), treatment (all treatment groups of zilurgisertib and placebo), and time‐by‐treatment interaction as fixed effects; and baseline QTc as a continuous covariate. Data from participants dosed with placebo were analyzed as a pooled group. Means of ECG and nominal times were used in all cases. An unstructured covariance matrix was specified for the repeated measures at post‐dose time points for participants within the treatment period. From this analysis, least squares mean (LSM) and two‐sided 90% CI were calculated for the contrast of “zilurgisertib vs placebo” for each dose of zilurgisertib and each post‐dose time point. For HR, PR, and QRS intervals, analyses were based on Δ post dose (ΔHR, ΔPR, and ΔQRS).

### Concentration‐ΔQTcI analysis

The exposure–response analysis strategy was based on recommendations by the scientific White Paper on C‐QTc modeling.^6^ A pre‐specified LME C‐QTc model was used to characterize the relationship between zilurgisertib plasma concentrations and ΔQTcI, with ΔQTcI as the dependent variable. Fixed effect parameters were intercept, slope, influence of baseline QTc (centered‐by‐mean) on intercept, treatment (active = 1 or placebo = 0), and nominal time with respect to the single dose administration. Plasma concentration of zilurgisertib (0 for placebo) was a continuous covariate. Participant was included as an additive random effect on both intercept and slope terms. It was assumed the random effects were normally distributed as a bivariate normal random variable, with mean (0, 0) and a 2 × 2 unstructured covariance matrix G, whereas the residuals were normally distributed with mean 0 and variance R. Degrees of freedom estimates were determined by the Kenward–Roger method.^10^

The following basic assumptions of the model were assessed using exploratory graphics: assumption that QTc interval is independent of HR, assumption of no time delay between drug concentration and ΔQTc, and assumption of a linear C‐ΔQTc relationship.

### Sensitivity and bias analyses

The effect of food on QTc was used to demonstrate ECG assay sensitivity, because administration of food shortens the QTc by 5‐10 ms, with the maximum effect occurring approximately 2‐4 hours after a meal.^11^ Using mean ECG readings and time‐matched PK concentration data collected from the 12 participants in cohort 9 (SAD study), a similar model to that used for the primary C‐QTc analysis was fitted with the main effects of treatment (active vs placebo) replaced with the main effect of the meal status with the addition of interactions between meal status and time. Assay sensitivity was shown by examining the contrasts of time fed–time fasted 2 hours and 4 hours after dosing. The Hochberg method^12^ was used to adjust for multiplicity because more than one post‐prandial time was analyzed. Assay sensitivity was assumed if the CI was completely <0 for at least one of the two time points considered.

A bias evaluation^13^ was performed to demonstrate that no bias was introduced by study cardiologist overreading at a level that would significantly affect the results. The relationship between the means and differences of the two ECG assessment methods (machine readings vs EPQT) was investigated by a robust regression using an M estimator to reduce the impact of individual outlier values.^14^

## Results

### Participant demographics and characteristics

Overall, 91 participants in the SAD study and 79 participants in the MAD study provided at least one pair of zilurgisertib PK and ECG data. In the SAD study, most participants (63.7%) were female, whereas in the MAD study, the majority (67.1%) were male (Table S2).

In general, ECG parameters at baseline varied within expected values for each dose group in both studies, and no participants had baseline values exceeding thresholds for more than two ECG parameters (Table S3).

### Central tendency analysis

LSMs (90% CI) of ΔΔHR were higher than ±5 (1.84, 12.50) bpm at 6, 8, and 12 hours after study drug administration for the 500‐mg dose group in the SAD study; higher than ±5 (3.56, 9.36) bpm at 4 hours after administration on day 1 of the MAD study for the 300‐mg BID dose group; and at 4 and 6 hours after administration on day 10 of the MAD study for 300 mg BID dose group. Dose‐dependent effects were observed for HR from the 10‐mg to 500‐mg dose groups in the SAD study (Figure S1a), from the 50‐mg QD to 300‐mg BID dose groups on day 1 and day 10 of the MAD study (Figure S1b).

Due to the violation of the assumption of no drug effect on HR in both studies, the QTcI QT correction method was evaluated as the primary QTc endpoint. Appropriateness of QTcI was assessed to determine if the assumption that QTcI was independent of HR (or RR) was valid, based on drug‐free data using an LME model with random effects on intercept. The mean (range) of QTcI slope coefficient (β_i_) for both studies combined was 0.20 (0.04, 0.40). In both studies, there appeared to be a positive correlation between QT and RR, a slightly negative correlation between QTcF and RR, and a flat trend between QTcI and RR (Table S4). Exploratory joint plots of the time courses of mean zilurgisertib plasma concentration, LSM of ∆∆QTcI and LSM of ∆∆HR across nominal time points up to 24 hours showed that there were no time‐delayed effects of zilurgisertib exposures on QTcI or HR (Figures S2‐S4). LSM (90% CI) absolute QTcF in SAD and MAD studies are shown in Figure S5.

**Figure 1:**
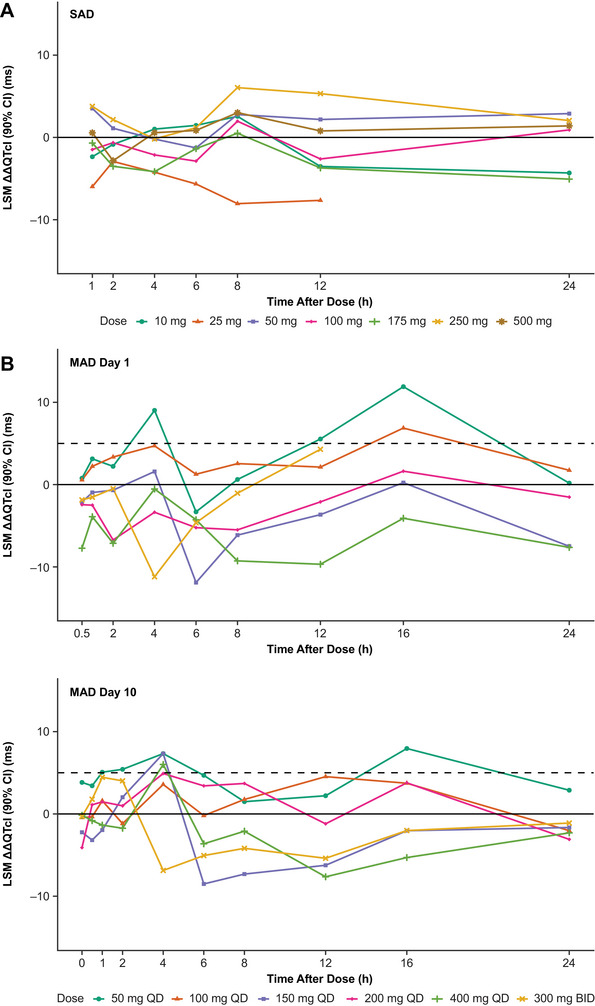
LSM (90% CI) ΔΔQTcI in (A) the single‐ascending dose (SAD) study and (B) the multiple‐ascending dose (MAD) study. ΔΔQTcI, placebo‐adjusted change from baseline in individualized heart rate–corrected QT interval; BID, twice daily; LSM, least squares mean; QD, once daily.

LSMs of ∆∆QTcI across escalating dose groups and at all‐time points up to 24 hours after administration were between −8.05 and 6.05 ms in the SAD study, between −11.9 and 11.9 ms on day 1 of the MAD study, and between −8.51 and 7.95 ms on day 10 of the MAD study. In the SAD study, the highest upper limit of 90% CIs of the LSM ∆∆QTcI was 12.3 ms in the dose group of 250 mg at the 8 hours post treatment timepoint. On day 1 of the MAD study, the highest upper limit of 90% CIs of the LSM ∆∆QTcI was 17.4 ms in the dose group of 50 mg QD at the 16 hour post‐dosing timepoint; on day 10, the highest upper limit of 90% CIs of the LSM ∆∆QTcI was 16.4 ms in the dose group of 50 mg QD at the 16 hour post‐dosing timepoint. The findings indicated there were no dose‐dependent or concentration‐dependent effects of zilurgisertib on QTcI (Figure 1). The placebo data represented as LSM (90% CI) ΔHR and ΔQTcI are shown in Figures S6 and S7.

In both studies, LSMs for ∆∆PR varied within ±5 ms across all dose groups for most time points up to 24 hours after administration. In addition, LSMs for ∆∆QRS were all small (within ±5 ms) across all dose groups and for all time points up to 24 hours after administration (data not shown).

### C‐ΔQTcI analysis

**Figure 2:**
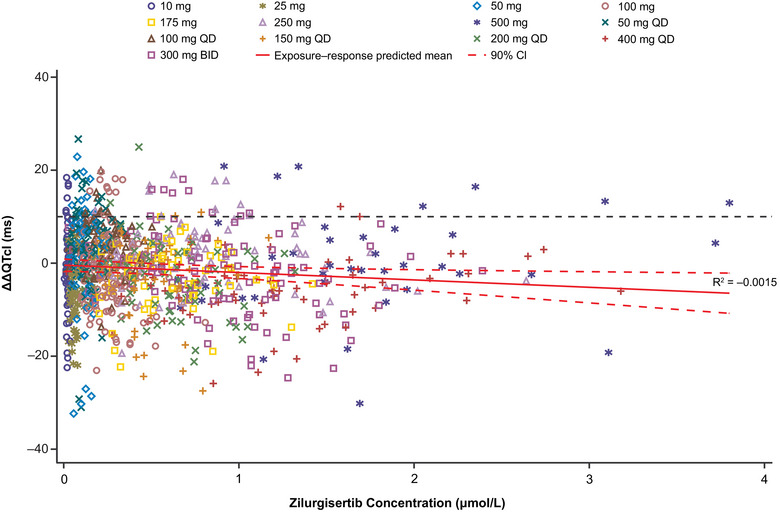
Observed zilurgisertib plasma concentrations and ΔΔQTcI with model prediction from the C‐QTcI linear mixed effects model estimates using pooled data from the single‐ascending dose study and the multiple‐ascending dose study (PK/QTc population). Black dashed line indicates threshold of clinically relevant QT effects (10 ms). Unit conversion factor between µm and µg/mL is 1 µm = 0.503 µg/mL. ΔΔQTcI, placebo‐adjusted change from baseline in individualized heart rate–corrected QT interval; BID, twice daily; C‐QTcI concentration‐individualized heart rate–corrected QT interval; PK, pharmacokinetic; QD, once daily; QTc, heart rate–corrected QT interval.

The final concentration‐ΔQTcI (C‐ΔQTcI) analysis was performed with pooled data from both studies (days 1 and 10). Furthermore, C‐ΔQTcI data from both studies could be adequately fitted by the pre‐specified LME models separately. Appropriateness of a linear relationship was assessed using a scatter plot of ΔΔQTcI and plasma concentrations for the pooled data overlaid with the exposure–response model‐predicted mean and 90% CI, in which the trend of the exposure–response model‐predicted means were similar (Figure 2). In addition, a decile plot of ΔΔQTcI and plasma concentrations overlaid with the exposure–response model‐predicted mean and 90% CI indicated the linear model was a reasonable fit to the data, either within each study/visit or the pooled data (Figure S8).

C‐ΔQTcI model parameter estimates and 90% CIs are presented in Table 1. The estimated population slope of the C‐ΔQTcI relationship was shallow at 0.02 ms per µm (standard error [SE], 0.319; 90% CI, −0.60, 0.65), which was not statistically significantly different from 0, and treatment effect–specific intercept was also small and non‐significant (−0.83 ms [SE, 0.728]; 90% CI, −2.26, 0.61).

**Table 1:** Parameter Estimates for the Final C‐ΔQTcI Model for Zilurgisertib

		All combined	
−2LL		14,143.0	
AIC		14,147.0	
AICc		14,147.0	
BIC		14,153.2	

Parameters	Estimate	SE	90% CI
Treatment (ms)	−0.828	0.728	(−2.26, 0.61)
Slope on concentration (ms/µm)	0.0244	0.319	(−0.60, 0.65)
Day 1			
0.5‐hour effect (ms)	−4.42	0.957	(−6.30, −2.54)
1‐hour effect (ms)	−3.94	0.687	(−5.29, −2.60)
2‐hour effect (ms)	−3.77	0.690	(−5.12, −2.41)
4‐hour effect (ms)	−1.59	0.685	(−2.93, −0.24)
6‐hour effect (ms)	−10.2	0.692	(−11.6, −8.86)
8‐hour effect (ms)	−10.4	0.697	(−11.8, −9.05)
12‐hour effect (ms)	−7.84	0.706	(−9.23, −6.46)
16‐hour effect (ms)	7.30	1.09	(5.17, 9.44)
24‐hour effect (ms)	−2.57	0.772	(−4.09, −1.06)
Day 10			
Pre effect (ms)	1.43	0.947	(−0.42, 3.29)
0.5‐hour effect (ms)	−4.32	0.955	(−6.19, −2.44)
1‐hour effect (ms)	−2.64	0.967	(−4.53, −0.74)
2‐hour effect (ms)	−2.40	0.984	(−4.33, −0.47)
4‐hour effect (ms)	−1.67	0.980	(−3.60, 0.25)
6‐hour effect (ms)	−10.6	0.959	(−12.5, −8.72)
8‐hour effect (ms)	−7.48	0.958	(−9.36, −5.60)
12‐hour effect (ms)	−9.98	0.945	(−11.8, −8.12)
16‐hour effect (ms)	2.87	0.942	(1.02, 4.72)
24‐hour effect (ms)	−4.82	0.942	(−6.67, −2.97)
Slope on centered baseline QTcI (no unit)	−0.0528	0.0143	(−0.08, −0.02)

Variance components		Estimate (95% CI)	
Variance (intercept)		35.3 (26.9, 48.4)	
Residual variance		60.6 (56.9, 64.7)	

−2LL, negative two times the log‐likelihood; AIC, Akaike information criterion; AICc, Akaike information criterion with a correction for small sample sizes; BIC, Bayesian information criterion; C‐ΔQTcI, concentration‐change from baseline in individualized heart rate–corrected QT interval; CI, confidence interval; QTcI, individualized heart rate–corrected QT interval; SE, standard error.

**Figure 3:**
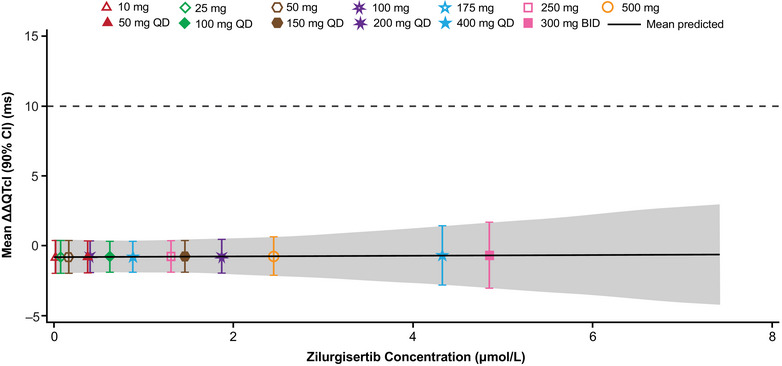
C‐ΔQTcI model‐predicted mean ΔΔQTcI (90% CI) at observed geometric mean peak zilurgisertib plasma concentrations. Unit conversion factor between µm and µg/mL is 1 µm = 0.503 µg/mL. ΔΔQTcI, placebo‐adjusted change from baseline in individualized heart rate–corrected QT interval; BID, twice daily; C‐ΔQTcI, concentration‐change from baseline in individualized heart rate–corrected QT interval; QD, once daily.

Based on the C‐ΔQTcI model, mean QT effect (ΔΔQTcI) was predicted to be −0.83 ms (90% CI, −2.02, 0.37) at 32.0 nm (or 16 ng/mL), the observed geometric mean of peak zilurgisertib concentrations with the lowest dose level studied (10 mg), and −0.71 ms (90% CI, −2.99, 1.57) at 4690 nm (or 2359 ng/mL), the highest observed geometric mean of peak zilurgisertib plasma concentrations after 300 mg BID (Figure 3). At no dose level did the upper limit of the two‐sided 90% confidence band of the predicted QT effect exceed 5 ms across the observed range of zilurgisertib concentrations in this study (i.e. up to 7420 nm [or 3732 ng/mL]).

### ECG categorical analysis

Across both studies, there was no apparent dose dependence in incidences of absolute QTcI >450 ms, ∆QTcI interval outliers, and HR >100 beats per min (bpm). With respect to the total number of participants and observations across all dose groups, in the SAD study (day 1) and for days 1 and 10 of the MAD study, incidence rates were ≤0.5% for absolute QTcI >450 ms, ≤2.2% for ∆QTcI interval outliers, and ≤4.1% for HR >100 bpm (Table S5‐S10).

Zilurgisertib did not have a clinically relevant effect on cardiac conduction in either study. All participants except for 2 in the SAD study had baseline and post‐treatment PR ≤200 ms. There was no dose dependence in incidence of QRS >100 ms.

### ECG assay sensitivity analysis

In the assay sensitivity analysis by food effect (cohort 9 of the SAD study), estimated effects of a high‐fat breakfast on LSM (90% CI) of ΔQTcI were −11.7 (−15.59, −7.81) ms and −8.6 (−12.46, −4.76) ms at 2 and 4 hours post dose. Estimated 90% CIs for these effects were <0, and thus ECG assay sensitivity was considered to have been established.

### ECG method bias analysis

In a comparison between fully automated machine readings of QTcF and EPQT readings, all Bland–Altman slopes were within the suggested threshold of ±0.10 in the absolute scale; therefore, minimal bias between the two ECG methods was demonstrated in both studies (Table S11).

## Discussion

It has been 20 years since the implementation of the ICH E14 clinical guidance specifying that all new drugs should undergo evaluation of QTc via thorough QT/QTc studies.^15^ Since then, the guidelines have been revised to allow the use of C‐QTc analysis for this purpose,^16^ an approach that can provide a fuller understanding of risk vs the more traditional thorough QT (TQT) analysis.^17^, ^18^ Potential benefits include: the opportunity to identify QT risks earlier, due to utilization of data from first‐in‐human studies vs a TQT study conducted later in the development process; clearer communication of risk compared to the “positive”/“negative” result given by TQT analyses, and providing the necessary safety data in a more cost‐effective manner than TQT.^17^, ^18^ Additionally, since the approach establishes the relationship with concentration, C‐QTc has the ability to provide QT projections for populations that may experience concentration profiles different from those experienced by the healthy subjects in a TQT analysis.^17^, ^18^

As outlined in the ICH E14/S7B Q&A guidance,^19^ replacement of a TQT study with C‐QTc analysis is considered appropriate when two key criteria are met: (1) a nonclinical package including hERG and in vivo QT studies demonstrates low proarrhythmic risk, and (2) clinical data show that a QTc effect exceeding 10 ms can be excluded with high confidence at clinically relevant and supratherapeutic exposures, typically defined as the upper bound of the two‐sided 90% CI for the predicted QTc effect being <10 ms. Nonclinical studies for zilurgisertib indicated low risk of QT prolongation. This analysis includes data from a SAD study (10‐500 mg) and a MAD study (10‐400 mg QD and 300 mg BID) evaluating zilurgisertib, and was conducted in place of a thorough QT study.

Initially, this analysis specified a primary endpoint of change from baseline in QT interval corrected by Fridericia's method (ΔQTcF), based on the assumption that there was no drug effect on HR. However, the central tendency analyses found that LSM of ΔΔHR was higher than ±5 bpm at 8 hours post study drug administration for the 500‐mg dose group in the SAD study, and at 4 hours post study drug administration for the 300‐mg BID dose group on both days 1 and 10 in the SAD study. Additionally, both studies indicated a dose‐dependent effect of zilurgisertib on HR. Given the assumption was not valid, QTcI was derived using a pre‐specified method and was used as the primary QTc endpoint data in lieu of QTcF for both studies.

The ICH E14 guidance and literature on C‐QTc modeling support the use of individualized corrections such as QTcI if drug‐free QT data are collected for all participants over a HR range similar to that observed during treatment.^6^, ^16^ In this analysis, both baseline and post‐baseline HR values were available for all 170 participants; the range of baseline HR values observed across both studies in this analysis was 38.4‐90.4 bpm (data not shown). Furthermore, the correlation analysis demonstrated that QTcI adequately corrected for HR, and exploratory joint plots showed that there were no time‐delayed effects of zilurgisertib exposures on QTcI or HR. In addition, QTcI has been used in a number of previous QT analyses.^20^, ^21^

Pooled data were used for the C‐ΔQTcI model based on aligned PK sampling, ECG methodology, and core lab procedures. The linear model fit the data well, with a non‐significant slope and intercept. The predicted ΔΔQTcI at the highest zilurgisertib concentration (300 mg BID; 7.4 µm [or 3.7 µg/mL]) was −0.71 ms, well below the regulatory concern threshold of 10 ms.

Overall, observed changes in ECG parameters from both studies were generally small. The categorical analysis did not reveal dose dependence in incidences of outliers in QTcI interval, HR, PR interval, and QRS interval. In the central tendency analysis, there were no dose‐dependent or concentration‐dependent effects of zilurgisertib on QTcI, and across both studies, the lowest LSM of ∆∆QTcI was −11.9 ms and the highest was 11.9 ms. Furthermore, LSMs for ∆∆PR and ∆∆QRS varied within ±5 ms across all dose groups for most time points up to 24 hours after administration.

Zilurgisertib is being evaluated at a dose of 100 mg QD in the ongoing phase 2, randomized, double‐blind, placebo‐controlled study in participants with FOP (ClinicalTrials.gov, NCT05090891). The mean observed zilurgisertib plasma concentrations at 100 mg QD (0.61 µm [or 0.3 µg/mL]) are expected to be approximately 12‐fold lower than the highest concentration (7.4 µm [or 3.7 µg/mL]) studied in this analysis and thus no cardiac safety concerns are anticipated. The supratherapeutic concentration of 7.4 µm (or 3.7 µg/mL) exceeds exposures observed under high clinical exposure scenarios, including those seen in dedicated renal and hepatic impairment studies (up to 70% and 25% higher AUC, respectively [data on file, Incyte]) and in the presence of strong CYP3A inhibition (2.5‐fold increase in AUC).^22^ These results support that the exposure–response analysis adequately covers worst‐case clinical scenarios in line with the 2022 ICH E14/S7B Q&A guidance.^19^ In addition, the hERG IC_50_ for zilurgisertib exceeds 100 µm (or 50 µg/mL), providing a >160‐fold margin over clinical C_max_ and ≥13‐fold over the highest concentration tested. Given the results of the present analysis, the waiver for thorough QT study has been approved by the FDA.

## Conclusions

There was no significant relationship between zilurgisertib plasma concentration and change in QTcI, and zilurgisertib did not have a clinically relevant effect on QTcI prolongation. Moreover, zilurgisertib did not have a relevant effect on cardiac conduction within the dose range studied (up to 300 mg BID). A QT effect exceeding the threshold of concern (10 ms) can be excluded for zilurgisertib plasma concentrations up to 7.4 µm (or 3.7 µg/mL).

## Conflicts of Interest

Yan‐ou Yang, Xiaohua Gong, Jay Getsy, and Kevin Rockich are employees of and own stock in Incyte Corporation. Hong Yang and Xing Liu are former employees of Incyte Corporation.

## Funding Information

This study was funded by Incyte Corporation (Wilmington, DE, USA).

## Supporting information



Supporting Information

## Data Availability

Incyte Corporation (Wilmington, DE, USA) is committed to data sharing that advances science and medicine while protecting patient privacy. Qualified external scientific researchers may request anonymized datasets owned by Incyte for the purpose of conducting legitimate scientific research. Researchers may request anonymized datasets from any interventional study (except phase 1 studies) for which the product and indication have been approved on or after January 1, 2020, in at least one major market (e.g. US, EU, JPN). Data will be available for request after the primary publication or 2 years after the study has ended. Information on Incyte's clinical trial data‐sharing policy and instructions for submitting clinical trial data requests are available at: https://www.incyte.com/Portals/0/Assets/Compliance%20and%20Transparency/clinical‐trial‐data‐sharing.pdf.
