# Supplementary material for: Evaluation of clinical cardiac safety of zilurgisertib, an activin receptor‐like kinase‐2 (ALK2) inhibitor, in healthy participants
Source: Clin Pharmacol Drug Dev. 2025 Oct 23;15(2):e1618. doi: 10.1002/cpdd.1618 (PMC12856974; doi:10.1002/cpdd.1618)

Supplementary Online Material

Evaluation of Clinical Cardiac Safety of Zilurgisertib, an activin receptor-like kinase-2 (ALK2) Inhibitor, in Healthy Participants

Yan-ou Yang, Hong Yang, Xing Liu, Xiaohua Gong, Jay Getsy, Kevin Rockich

Incyte Corporation, Wilmington, DE, USA

Corresponding author:
Yan-ou Yang
Clinical Pharmacology and Pharmacometrics,
Incyte Corporation, 1801 Augustine Cut Off, Wilmington, DE 19803
Tel: 302-605-0184; E-mail: yyang@incyte.com

Table S1. ECG Categorical Analysis

ECG Parameter	ECG Category	Baseline ECG Stratification
QTc	Value ≤ 450 ms<br>450 ms < value ≤ 480 ms<br>480 ms < value ≤ 500 ms<br>Value > 500 ms	Baseline QTc ≤ 450 ms vs > 450 ms
ΔQTc	Value ≤ 30 ms<br>30 ms < value ≤ 60 ms<br>Value > 60 ms	Baseline QTc ≤ 450 ms vs > 450 ms
HR	Value ≤ 100 bpm<br>Value > 100 bpm	Baseline HR ≤ 100 bpm vs > 100 bpm
PR	Value ≤ 200 ms<br>200 ms < value ≤ 220 ms<br>Value > 220 ms	Baseline PR ≤ 200 ms vs > 200 ms
QRS	Value ≤ 100 ms<br>100 ms < value ≤ 110 ms<br>Value > 110 ms	Baseline QRS ≤ 100 ms vs > 100 ms

ΔQTc, change from baseline in heart rate–corrected QT interval; bpm, beats per min; ECG, electrocardiogram; HR, heart rate; PR, time from the beginning of the P wave to the beginning of the next QRS complex; QRS, deflections in the tracing of the electrocardiogram comprising the Q, R, and S waves that represent depolarization of the ventricles; QTc, QT interval corrected for heart rate.

Table S2. Summary of the Analysis Populations

Characteristic	PK Population	ECG Population	C-ΔQTc Population
SAD study			
Number of participants, n	70	91	91
Male, n (%)	24 (34.3)	33 (36.3)	33 (36.3)
Zilurgisertib dose group, n (%)			
Placebo	0	21 (23.1)	21 (23.1)
10 mg	9 (12.9)	9 (9.9)	9 (9.9)
25 mg	9 (12.9)	9 (9.9)	9 (9.9)
50 mg	9 (12.9)	9 (9.9)	9 (9.9)
100 mg	19 (27.1)	19 (20.9)	19 (20.9)
175 mg	9 (12.9)	9 (9.9)	9 (9.9)
250 mg	9 (12.9)	9 (9.9)	9 (9.9)
500 mg	6 (8.6)	6 (6.6)	6 (6.6)
MAD study			
Number of participants, n	59	79	79
Male, n (%)	38 (64.4)	53 (67.1)	53 (67.1)
Zilurgisertib dose group, n (%)			
Placebo	0	20 (25.3)	20 (25.3)
50 mg QD	9 (15.3)	9 (11.4)	9 (11.4)
100 mg QD	9 (15.3)	9 (11.4)	9 (11.4)
150 mg QD	9 (15.3)	9 (11.4)	9 (11.4)
200 mg QD	9 (15.3)	9 (11.4)	9 (11.4)
400 mg QD	9 (15.3)	9 (11.4)	9 (11.4)
300 mg BID	14 (23.7)	14 (17.7)	14 (17.7)

BID, twice daily; C-ΔQTc, concentration-change from baseline in heart rate–corrected QT interval; ECG, electrocardiogram; MAD, multiple ascending dose; PK, pharmacokinetic; QD, once daily; SAD, single ascending dose.

Table S3. Baseline Electrogram Strata

Single-ascending dose study						
StrataQTcF (ms)	StrataQTcI (ms)	StrataHR (beats/min)	StrataPR (ms)	StrataQRS (ms)	Number of Participants	Percentage (n=91)
≤ 450	≤ 450	≤ 100	≤ 200	≤ 100	24	22.8%
≤ 450	≤ 450	≤ 100	≤ 200	> 100	59	69.4%
≤ 450	≤ 450	≤ 100	> 200	> 100	2	2.35%
**Multiple-ascending dose study**						
StrataQTcF (ms)	StrataQTcI (ms)	StrataHR (beats/min)	StrataPR (ms)	StrataQRS (ms)	Number of Participants	Percentage (n=79)
≤ 450	≤ 450	≤ 100	≤ 200	≤ 100	18	22.8%
≤ 450	≤ 450	≤ 100	≤ 200	> 100	60	75.9%
≤ 450	> 450	≤ 100	≤ 200	> 100	1	1.27%

HR, heart rate; PR, PR interval; QRS, QRS, interval; QTcF, heart rate–corrected QT interval by Fridericia's method; QTcI, individualized heart rate–corrected QT interval.

Table S4. Parameter Estimation of QT/QTc-RR Model

Study	Category	Estimate of Slope	*P* Value	Alpha	Lower	Upper	R^2
SAD study	QT vs RR	0.09125	< 0.0001	0.05	0.08777	0.09473	0.51244
	QTcF vs RR	−0.03909	< 0.0001	0.05	−0.04244	−0.03574	0.69892
	QTcI vs RR	−0.00080	0.6055	0.05	−0.00385	0.002245	0.68260
MAD study	QT vs RR	0.07682	< 0.0001	0.05	0.07351	0.08012	0.50339
	QTcF vs RR	−0.05206	< 0.0001	0.05	−0.05528	−0.04885	0.72705
	QTcI vs RR	−0.00044	0.7723	0.05	−0.00341	0.002531	0.67056

MAD, multiple ascending dose; QTc, heart rate–corrected QT interval; QTcF, heart rate–corrected QT interval by Fridericia's method; QTcI, individualized heart rate–corrected QT interval; RR, time elapsed between two consecutive R waves; SAD, single ascending dose.

Table S5. Categorical Analysis of QTcI or ΔQTcI by Dose Group (SAD Study)

Category	Placebo	10 mg	25 mg	50 mg	100 mg	175 mg	250 mg	500 mg	100 mg Fed	All Dose Groups
Baseline QTcI ≤ 450 ms*										
QTcI Interval										
QTcI total	21	9	9	9	19	9	9	6	12	103
QTcI ≤ 450 ms	21 (100%)	9 (100%)	9 (100%)	9 (100%)	19 (100%)	9 (100%)	9 (100%)	6 (100%)	12 (100%)	103 (100%)
QTcI > 450 and ≤ 480 ms	0	1 (11.1%)	0	0	0	0	0	0	0	1 (1.0%)
ΔQTcI										
ΔQTcI total	21	9	9	9	19	9	9	6	12	103
ΔQTcI ≤ 30 ms	21 (100%)	9 (100%)	9 (100%)	9 (100%)	19 (100%)	9 (100%)	9 (100%)	6 (100%)	12 (100%)	103 (100%)

*No participants had baseline QTcI > 450 ms.

Note: Values are presented as number of participants, n, or n (%) in which the frequency (%) was calculated with respect to the total count of the match parameters within each column. Categories with a value of 0 in all dose groups are not shown.

QTcI, individualized heart rate–corrected QT interval; SAD, single ascending dose.

Table S6. Categorical Analysis of QTcI or ΔQTcI by Dose Group (MAD Study, Day 1)

Category	Placebo	50 mg QD	100 mg QD	150 mg QD	200 mg QD	400 mg QD	300 mg BID	All Dose Groups
Baseline QTcI ≤ 450 ms								
QTcI Interval								
QTcI total	20	8	9	9	9	8	14	77
QTcI ≤ 450 ms	20 (100%)	8 (100%)	9 (100%)	9 (100%)	9 (100%)	8 (100%)	14 (100%)	77 (100%)
QTcI > 450 and ≤ 480 ms	1 (5.0%)	0	0	0	0	0	0	1 (1.3%)
ΔQTcI								
ΔQTcI total	20	8	9	9	9	8	14	77
ΔQTcI ≤ 30 ms	20 (100%)	8 (100%)	9 (100%)	9 (100%)	9 (100%)	8 (100%)	14 (100%)	77 (100%)
ΔQTcI > 30 and ≤ 60 ms	0	1 (12.5%)	0	0	0	0	0	1 (1.3%)
Baseline QTcI > 450 ms								
QTcI								
QTcI total	0	1	0	0	0	0	0	1
QTcI ≤ 450 ms	0	1 (100%)	0	0	0	0	0	1 (100%)
QTcI > 450 and ≤ 480 ms	0	1 (100%)	0	0	0	0	0	1 (100%)
ΔQTcI								
ΔQTcI total	0	1	0	0	0	0	0	1
ΔQTcI ≤ 30 ms	0	1 (100%)	0	0	0	0	0	1 (100%)

Note: Values are presented as number of participants, n, or n (%) in which the frequency (%) was calculated with respect to the total count of the match parameters within each column. Categories with a value of 0 in all dose groups are not shown.

BID, twice daily; MAD, multiple ascending dose; QD, once daily; QTcI, individualized heart rate–corrected QT interval.

Table S7. Categorical Analysis of QTcI or ΔQTcI by Dose Group (MAD Study, Day 10)

Category	Placebo	50 mg QD	100 mg QD	150 mg QD	200 mg QD	400 mg QD	300 mg BID	All Dose Groups
Baseline QTcI ≤ 450 ms								
QTcI Interval								
QTcI total	20	8	9	9	9	9	12	76
QTcI ≤ 450 ms	20 (100%)	8 (100%)	9 (100%)	9 (100%)	9 (100%)	9 (100%)	12 (100%)	76 (100%)
QTcI > 450 and ≤ 480 ms	1 (5.0%)	0	0	0	0	1 (11.1%)	0	2 (2.6%)
ΔQTcI								
ΔQTcI total	20	8	9	9	9	9	12	76
ΔQTcI ≤ 30 ms	20 (100%)	8 (100%)	9 (100%)	9 (100%)	9 (100%)	9 (100%)	12 (100%)	76 (100%)
ΔQTcI > 30 and ≤ 60 ms	0	1 (12.5%)	0	0	0	1 (11.1%)	0	2 (2.6%)
Baseline QTcI > 450 ms								
QTcI								
QTcI total	0	1	0	0	0	0	0	1
QTcI ≤ 450 ms	0	1 (100%)	0	0	0	0	0	1 (100%)
ΔQTcI								
ΔQTcI total	0	1	0	0	0	0	0	1
ΔQTcI ≤ 30 ms	0	1 (100%)	0	0	0	0	0	1 (100%)

Note: Values are presented as number of participants, n, or n (%) in which the frequency (%) was calculated with respect to the total count of the match parameters within each column. Categories with a value of 0 in all dose groups are not shown.

BID, twice daily; MAD, multiple ascending dose; QD, once daily; QTcI, individualized heart rate–corrected QT interval.

Table S8. Categorical Analysis of Heart Rate, PR, and QRS by Dose Group (SAD Study)

Category	Placebo	10 mg	25 mg	50 mg	100 mg	175 mg	250 mg	500 mg	100 mg Fed	All Dose Groups
HR										
Baseline HR ≤ 100 bpm*										
HR Total	21	9	9	9	19	9	9	6	12	103
HR ≤ 100 bpm	21 (100%)	9 (100%)	9 (100%)	9 (100%)	19 (100%)	9 (100%)	9 (100%)	6 (100%)	12 (100%)	103 (100%)
HR > 100 bpm	0	0	0	0	0	0	0	0	1 (8.3%)	1 (1.0%)
PR										
Baseline PR ≤ 200 ms										
PR Total	20	9	9	8	19	9	9	6	12	101
PR ≤ 200 ms	20 (100%)	9 (100%)	9 (100%)	8 (100%)	19 (100%)	9 (100%)	9 (100%)	6 (100%)	12 (100%)	101 (100%)
Baseline PR > 200 ms										
PR Total	1	0	0	1	0	0	0	0	0	2
PR ≤ 200 ms	1 (100%)	0	0	1 (100%)	0	0	0	0	0	2 (100%)
200 ms < PR ≤ 220 ms	1 (100%)	0	0	1 (100%)	0	0	0	0	0	2 (100%)
QRS										
Baseline QRS ≤ 100 ms										
QRS Total	4	4	3	0	7	3	1	2	2	26
QRS ≤ 100 ms	4 (100%)	4 (100%)	3 (100%)	0	7 (100%)	3 (100%)	1 (100%)	2 (100%)	2 (100%)	26 (100%)
100 ms < QRS ≤ 110 ms	0	0	0	0	3 (42.9%)	0	0	1 (50.0%)	0	4 (15.4%)
Baseline QRS > 100 ms										
QRS Total	17	5	6	9	12	6	8	4	10	77
QRS ≤ 100 ms	1 (5.9%)	0	1 (16.7%)	3 (33.3%)	1 (8.3%)	0	1 (12.5%)	0	2 (20.0%)	9 (11.7%)
100 ms < QRS ≤ 110 ms	16 (94.1%)	5 (100%)	6 (100%)	7 (77.8%)	10 (83.3%)	5 (83.3%)	6 (75.0%)	4 (100%)	8 (80.0%)	67 (87.0%)
QRS > 110 ms	3 (17.6%)	1 (20.0%)	1 (16.7%)	4 (44.4%)	3 (25.0%)	1 (16.7%)	2 (25.0%)	1 (25.0%)	3 (30.0%)	19 (24.7%)

*No participants had baseline HR >100 bpm.

Note: Values are presented as number of participants, n, or n (%) in which the frequency (%) was calculated with respect to the total count of the match parameters within each column. Categories with a value of 0 in all dose groups are not shown.

bpm, beats per minute; HR, heart rate; PR, PR interval; QRS, QRS, interval; SAD, single ascending dose.

Table S9. Categorical Analysis of Heart Rate, PR, and QRS by Dose Group (MAD Study, Day 1)

Category	Placebo	50 mg QD	100 mg QD	150 mg QD	200 mg QD	400 mg QD	300 mg BID	All Dose Groups
HR								
Baseline HR ≤ 100 bpm*								
HR Total	20	9	9	9	9	8	14	78
HR ≤ 100 bpm	20 (100%)	9 (100%)	9 (100%)	9 (100%)	9 (100%)	8 (100%)	14 (100%)	78 (100%)
HR > 100 bpm	0	0	0	0	0	0	1 (7.1%)	1 (1.3%)
PR								
Baseline PR ≤ 200 ms†								
PR Total	20	9	9	9	9	8	14	78
PR ≤ 200 ms	20 (100%)	9 (100%)	9 (100%)	9 (100%)	9 (100%)	8 (100%)	14 (100%)	78 (100%)
QRS								
Baseline QRS ≤ 100 ms								
QRS Total	2	3	3	2	2	1	5	18
QRS ≤ 100 ms	2 (100%)	3 (100%)	3 (100%)	2 (100%)	2 (100%)	1 (100%)	5 (100%)	18 (100%)
Baseline QRS > 100 ms								
QRS Total	18	6	6	7	7	7	9	60
QRS ≤ 100 ms	3 (16.7%)	3 (50.0%)	2 (33.3%)	1 (14.3%)	2 (28.6%)	1 (14.3%)	1 (11.1%)	13 (21.7%)
100 ms < QRS ≤ 110 ms	17 (94.4%)	5 (83.3%)	5 (83.3%)	6 (85.7%)	7 (100%)	5 (71.4%)	7 (77.8%)	52 (86.7%)
QRS > 110 ms	6 (33.3%)	2 (33.3%)	2 (33.3%)	2 (28.6%)	3 (42.9%)	4 (57.1%)	2 (22.2%)	21 (35.0%)

*No participants had baseline HR >100 bpm. †No participants had baseline PR >200 ms.

Note: Values are presented as number of participants, n, or n (%) in which the frequency (%) was calculated with respect to the total count of the match parameters within each column. Categories with a value of 0 in all dose groups are not shown.

BID, twice daily; HR, heart rate; MAD, multiple ascending dose; PR, PR interval; QRS, QRS, interval; QD, once daily.

Table S10. Categorical Analysis of Heart Rate, PR, and QRS by Dose Group (MAD Study, Day 10)

Category	Placebo	50 mg QD	100 mg QD	150 mg QD	200 mg QD	400 mg QD	300 mg BID	All Dose Groups
HR								
Baseline HR ≤ 100 bpm*								
HR Total	20	9	9	9	9	9	12	77
HR ≤ 100 bpm	20 (100%)	9 (100%)	9 (100%)	9 (100%)	9 (100%)	9 (100%)	12 (100%)	77 (100%)
HR > 100 bpm	0	0	0	0	0	0	2 (16.7%)	2 (2.6%)
PR								
Baseline PR ≤ 200 ms†								
PR Total	20	9	9	9	9	9	12	77
PR ≤ 200 ms	20 (100%)	9 (100%)	9 (100%)	9 (100%)	9 (100%)	9 (100%)	12 (100%)	77 (100%)
QRS								
Baseline QRS ≤ 100 ms								
QRS Total	2	3	3	2	2	1	4	21
QRS ≤ 100 ms	2 (100%)	3 (100%)	3 (100%)	2 (100%)	2 (100%)	1 (100%)	4 (100%)	17 (100%)
100 ms < QRS ≤ 110 ms	2 (100%)	0	0	1 (50.0%)	0	0	1 (25.0%)	4 (23.5%)
Baseline QRS > 100 ms								
QRS Total	18	6	6	7	7	8	8	60
QRS ≤ 100 ms	8 (44.4%)	1 (16.7%)	2 (33.3%)	1 (14.3%)	3 (42.9%)	1 (12.5%)	1 (12.5%)	17 (28.3%)
100 ms < QRS ≤ 110 ms	16 (88.9%)	4 (66.7%)	6 (100%)	7 (100%)	7 (100%)	5 (62.5%)	7 (87.5%)	52 (86.7%)
QRS > 110 ms	6 (33.3%)	3 (50.0%)	2 (33.3%)	3 (42.9%)	2 (28.6%)	4 (50.0%)	2 (25.0%)	22 (36.7%)

*No participants had baseline HR >100 bpm. †No participants had baseline PR >200 ms.

Note: Values are presented as number of participants, n, or n (%) in which the frequency (%) was calculated with respect to the total count of the match parameters within each column. Categories with a value of 0 in all dose groups are not shown.

BID, twice daily; HR, heart rate; MAD, multiple ascending dose; PR, PR interval; QRS, QRS, interval; QD, once daily.

Table S11. Summary of Bland–Altman Plot Parameters in the ECG Method Bias Analysis

SAD study		
Treatment	Mean (95% CI) of Differences (EPQT − Machine Generated) (ms)	Bland–Altman Slope Mean (95% CI) (ms)
Placebo	−0.49 (−0.84, −0.13)	0.00 (−0.01, 0.02)
10 mg	0.85 (−1.21, 2.91)	−0.004 (−0.02, 0.01)
25 mg	0.00 (−0.45, 0.45)	0.01 (−0.01, 0.04)
50 mg	0.093 (−0.41, 0.60)	0.03 (0.00, 0.06)
100 mg	−0.21 (−0.50, 0.08)	0.01 (−0.00, 0.03)
175 mg	0.19 (−0.32, 0.70)	−0.03 (−0.06, 0.01)
250 mg	0.28 (−0.31, 0.86)	0.01 (−0.02, 0.05)
500 mg	−0.16 (−0.85, 0.54)	−0.02 (−0.06, 0.02)
100 mg fed	0.46 (−0.94, 1.85)	−0.01 (−0.03, 0.01)
Overall	0.03 (−0.24, 0.30)	0.00 (−0.00, 0.01)
MAD study		
Treatment	Mean (95% CI) of Differences (EPQT – Machine Generated) (ms)	Bland–Altman Slope Mean (95% CI) (ms)
Placebo	1.33 (0.28, 2.38)	0.00 (−0.01, 0.01)
50 mg QD	−0.26 (−0.56, 0.05)	0.01 (−0.00, 0.02)
100 mg QD	−0.58 (−1.08, −0.08)	0.01 (−0.00, 0.03)
150 mg QD	0.01 (−0.74, 0.76)	0.04 (0.01, 0.06)
200 mg QD	0.51 (−1.09, 2.12)	0.05 (0.02, 0.07)
400 mg QD	−1.17 (−1.99, −0.36)	−0.003 (−0.02, 0.02)
300 mg BID	−0.15 (−1.07, 0.78)	0.01 (0.00, 0.03)
Overall	0.16 (−0.23, 0.55)	0.01 (0.01, 0.02)

BID, twice daily; CI, confidence interval; ECG, electrocardiogram; EPQT, Expert Precision QT assessment; MAD, multiple ascending dose; SAD, single ascending dose; QD, once daily.

Figure S1. LSM (90% CI) ΔΔHR in (a) the single-ascending dose (SAD) study and (b) the multiple-ascending dose (MAD) study. ΔΔHR, placebo-adjusted change from baseline in heart rate; bpm, beats per min; BID, twice daily; LSM, least squares mean; QD, once daily.

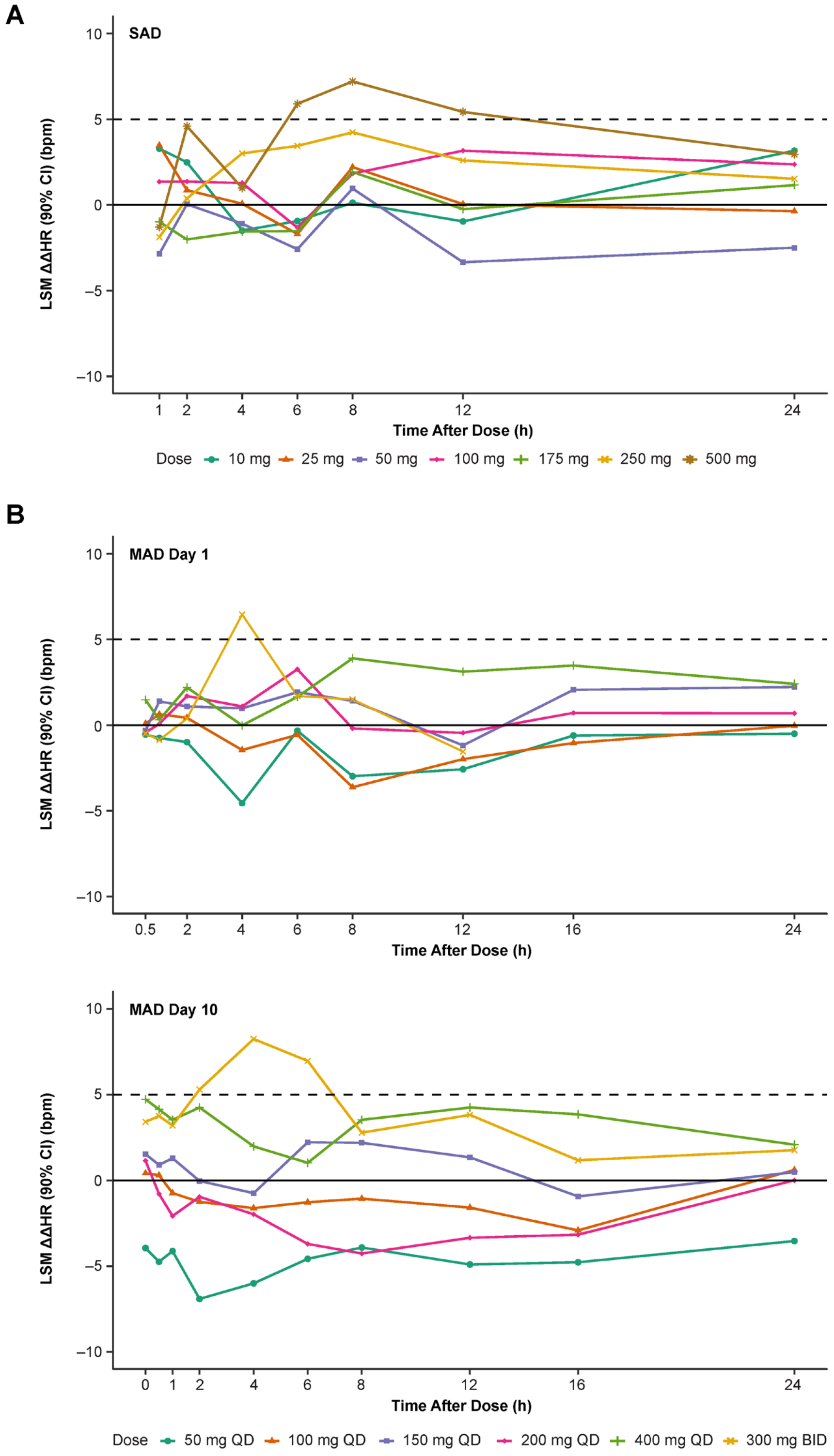

Figure S2. Joint plots of time-aligned mean plasma zilurgisertib concentration (90% CI), LSM ΔΔQTcI (90% CI), and LSM ΔΔHR (90% CI) in the single-ascending dose study. Unit conversion factor between μM and μg/mL is 1 μM = 0.503 μg/mL ΔΔHR, placebo-adjusted change from baseline in heart rate; ΔΔQTcI, placebo-adjusted change from baseline in individualized heart rate–corrected QT interval; LSM, least squares mean.

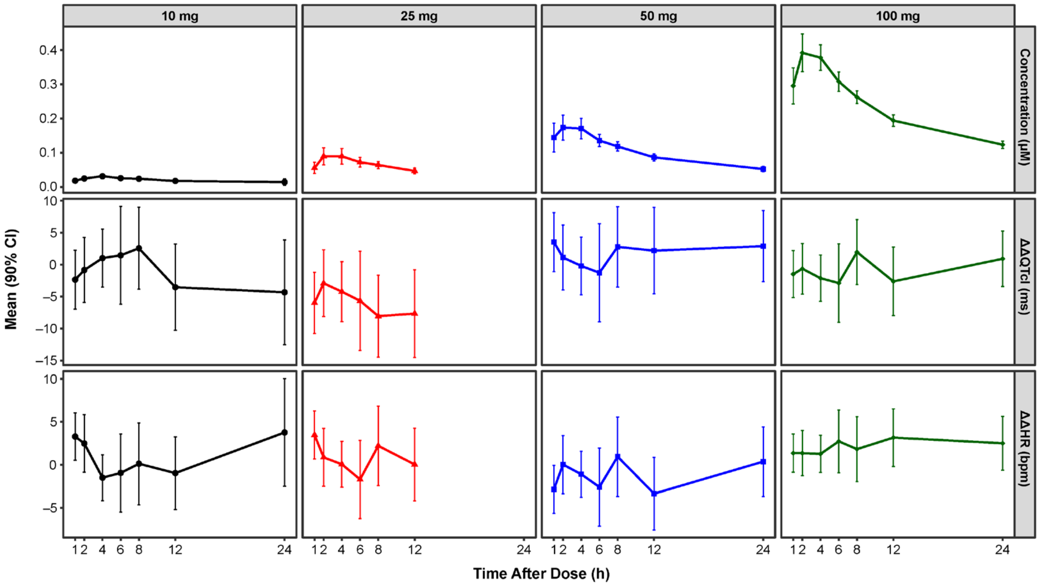

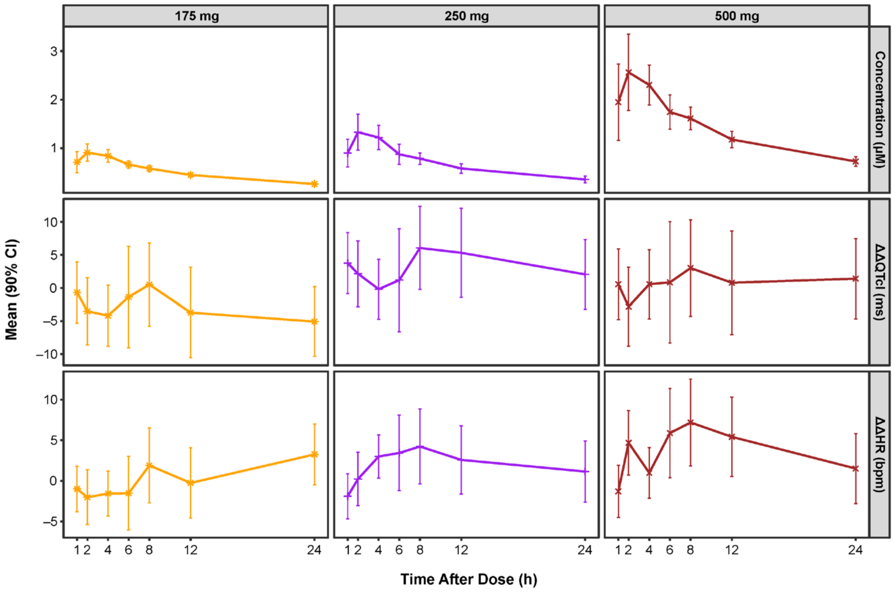

Figure S3. Joint plots of time-aligned mean plasma zilurgisertib concentration (90% CI), LSM ΔΔQTcI (90% CI), and LSM ΔΔHR (90% CI) in multiple-ascending dose study, day 1. Unit conversion factor between µM and µg/mL is 1 µM = 0.503 µg/mL. ΔΔHR, placebo-adjusted change from baseline in heart rate; ΔΔQTcI, placebo-adjusted change from baseline in individualized heart rate–corrected QT interval; BID, twice daily; LSM, least squares mean; QD, once daily.

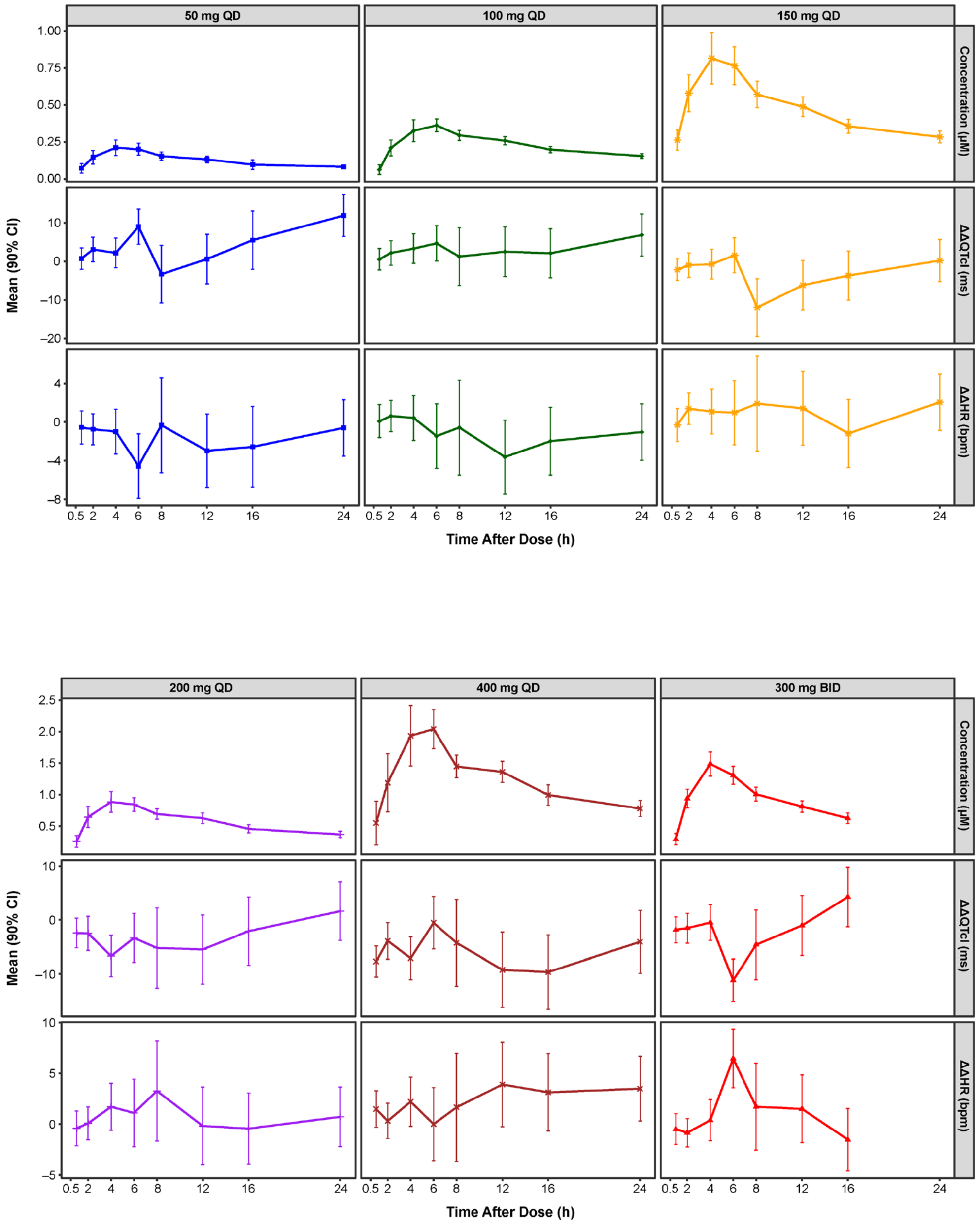

Figure S4. Joint plots of time-aligned mean plasma zilurgisertib concentration (90% CI), LSM ΔΔQTcI (90% CI), and LSM ΔΔHR (90% CI) in the multiple-ascending dose study, day 10. Unit conversion factor between μM and μg/mL is 1 μM = 0.503 μg/mL. ΔΔHR, placebo-adjusted change from baseline in heart rate; ΔΔQTcI, placebo-adjusted change from baseline in individualized heart rate–corrected QT interval; BID, twice daily; LSM, least squares mean; QD, once daily.

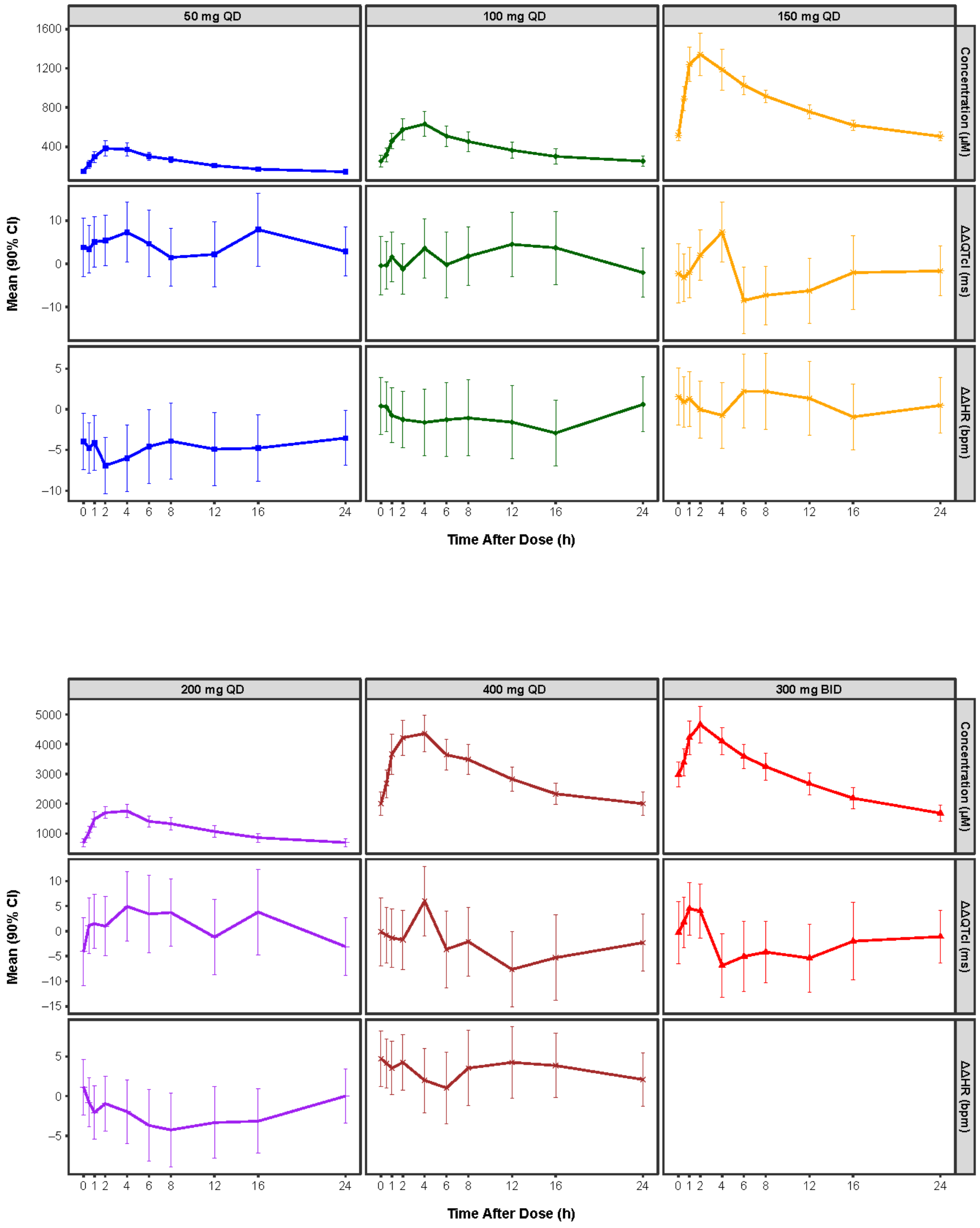

Figure S5. LSM (90% CI) absolute QTcF in (a) single-ascending dose (SAD) study INCB00928-101, (b) multiple-ascending dose (MAD) study. QTcF, Fridericia-corrected QT interval; ms, millisecond; BID, twice daily; LSM, least squares mean; QD, once daily.

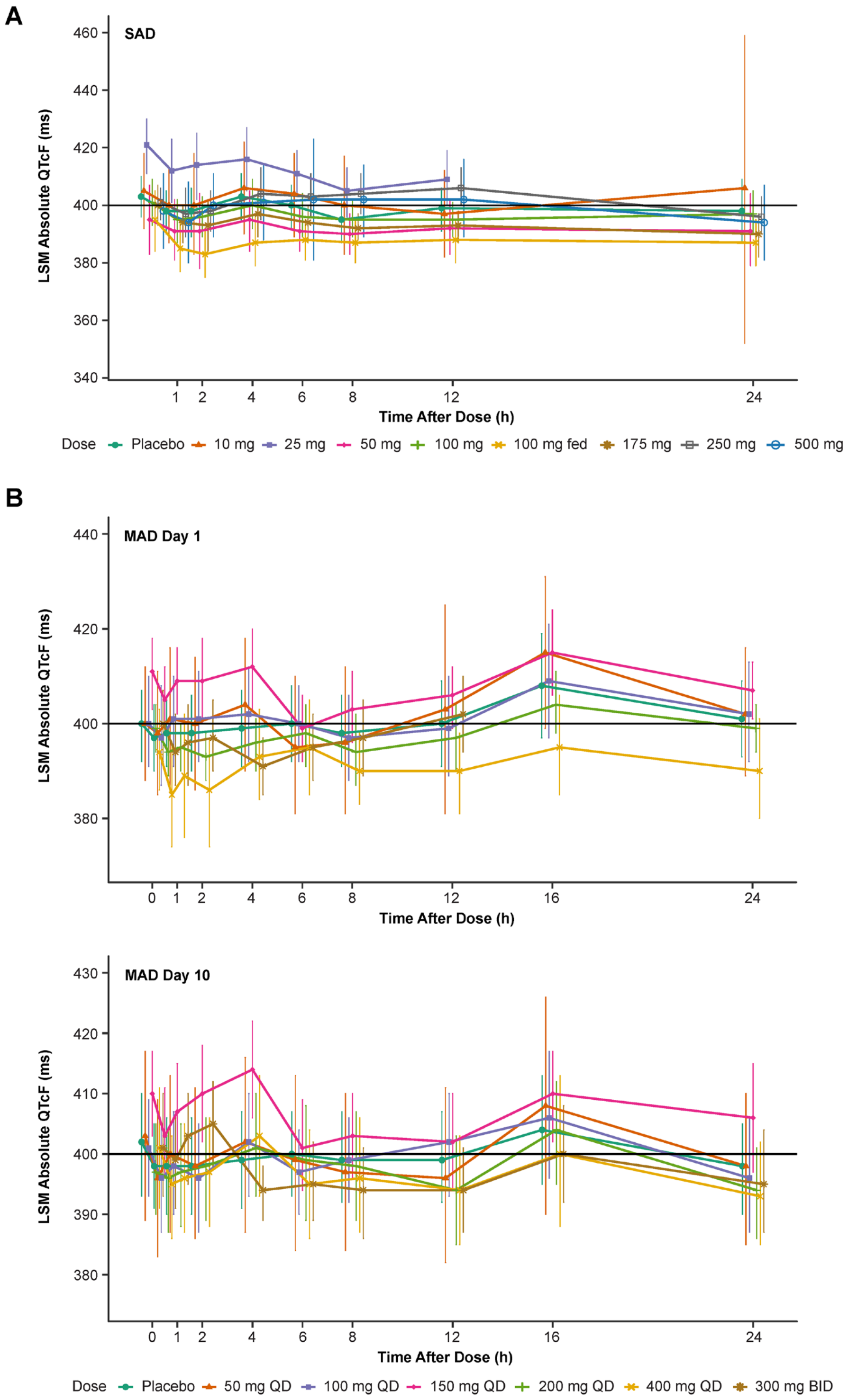

Figure S6. LSM (90% CI) ΔHR in in (a) single-ascending dose (SAD) study INCB00928-101, (b) multiple-ascending dose (MAD) study. ΔHR, change from baseline in heart rate; bpm, beats per min; BID, twice daily; LSM, least squares mean; QD, once daily.

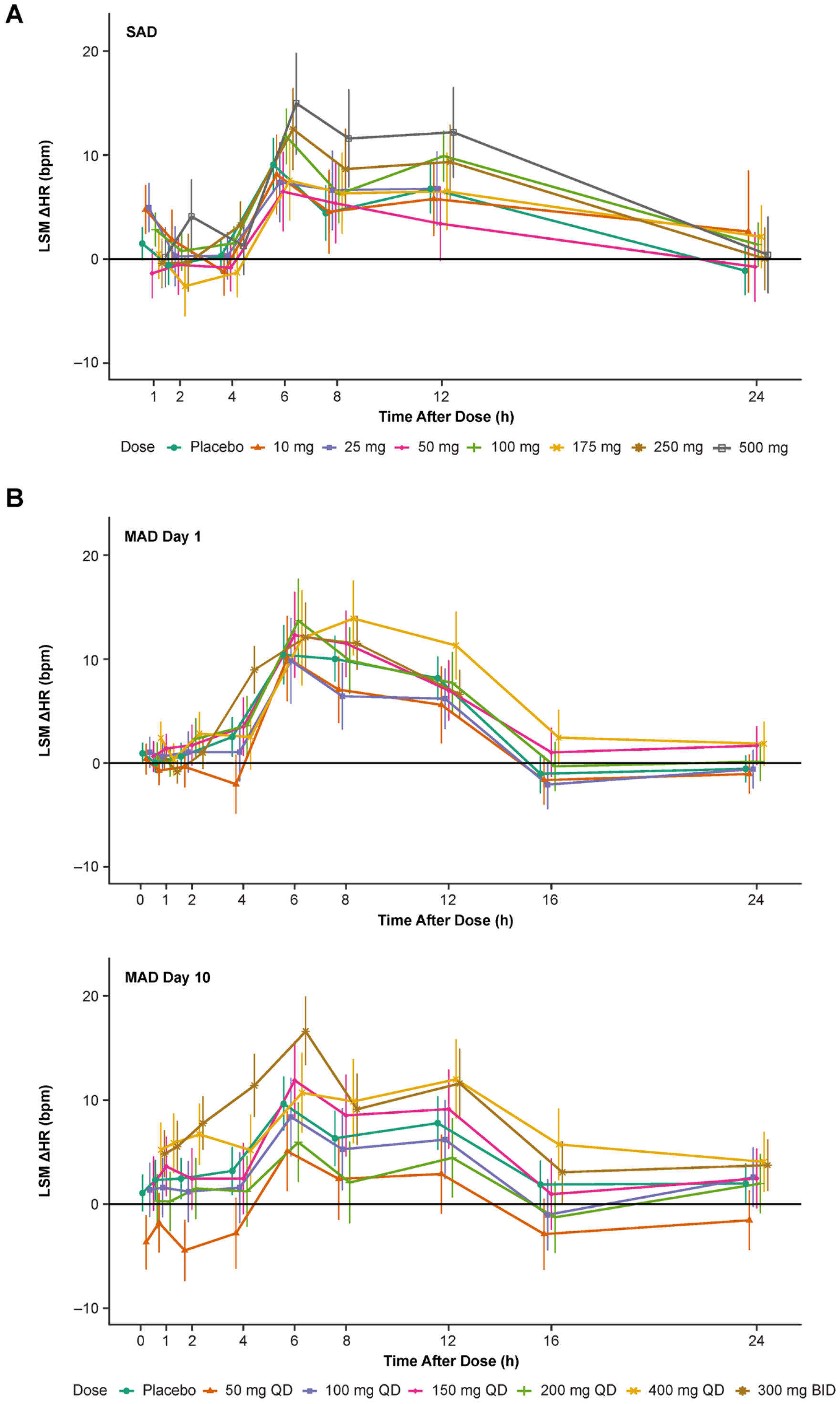

Figure S7. LSM (90% CI) ΔQTcI in (a) (a) single-ascending dose (SAD) study INCB00928-101, (b) multiple-ascending dose (MAD) study. ΔQTcI, change from baseline in individualized heart rate–corrected QT interval; ms, millisecond; BID, twice daily; LSM, least squares mean; QD, once daily.

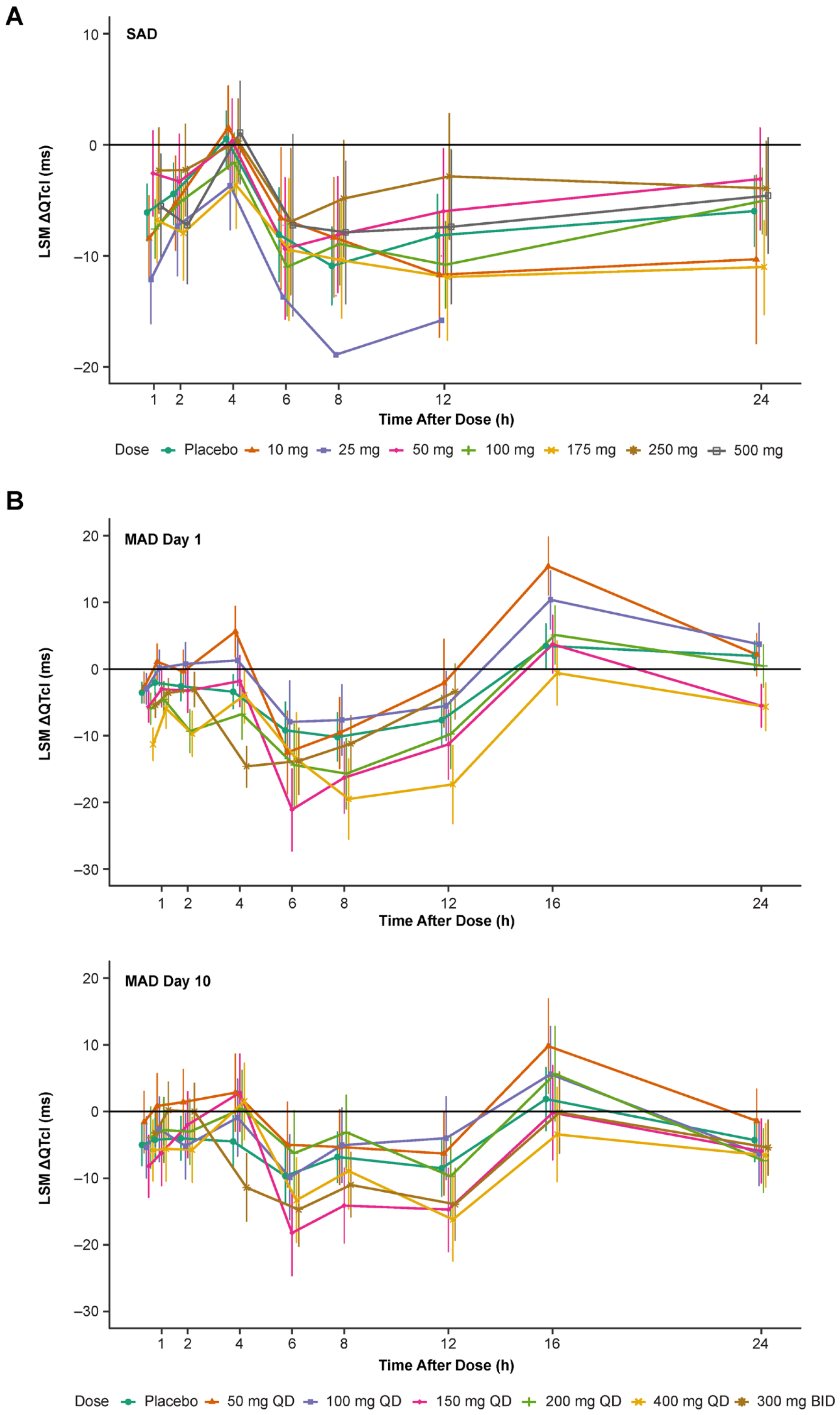

Figure S8. Model-predicted mean ΔΔQTcI (90% CI) and observed mean ΔΔQTcI (90% CI) across deciles of zilurgisertib plasma concentrations from C-QTcI linear mixed effects model estimates (PK/QTc population). Unit conversion factor between μM and μg/mL is 1 μM = 0.503 μg/mL. ΔΔQTcI, placebo-adjusted change from baseline in individualized heart rate–corrected QT interval; ΔΔQTc, placebo-adjusted heart rate–corrected QT interval; C-QTcI concentration-individualized heart rate–corrected QT interval; PK, pharmacokinetic; QTc, heart rate–corrected QT interval.

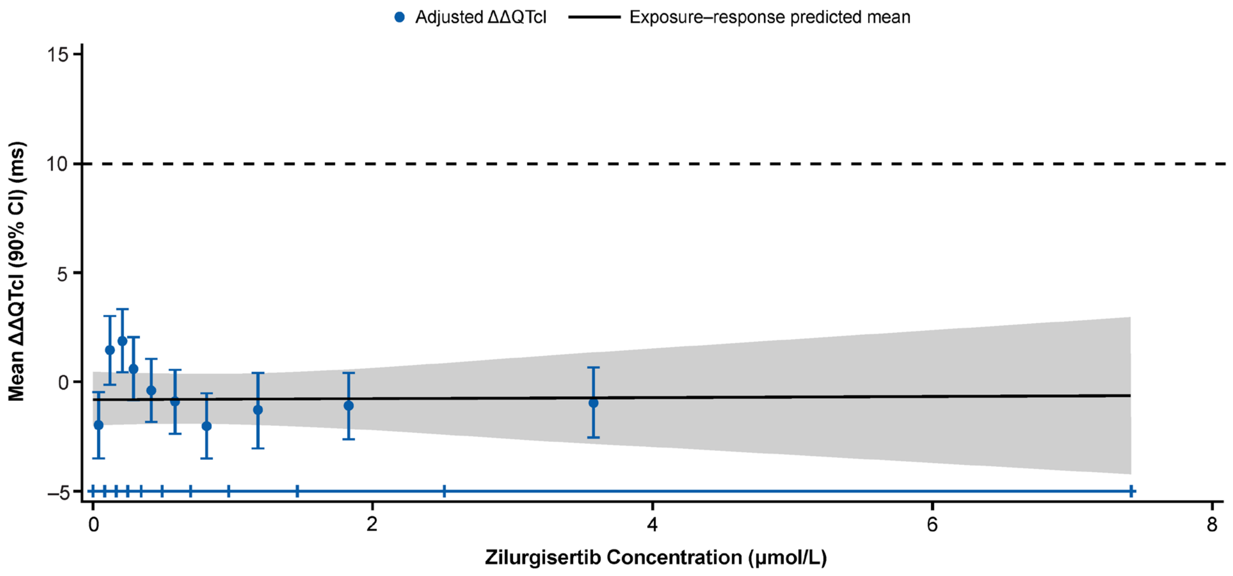

Supplement: Supplementary file 1 — Supporting Information [file CPDD-15-0-s001.pdf]
